# Dementia and COVID-19: A Case Report and Literature Review on Pain Management

**DOI:** 10.3390/ph15020199

**Published:** 2022-02-04

**Authors:** Damiana Scuteri, Marianna Contrada, Paolo Tonin, Maria Tiziana Corasaniti, Pierluigi Nicotera, Giacinto Bagetta

**Affiliations:** 1 Pharmacotechnology Documentation and Transfer Unit, Preclinical and Translational Pharmacology, Department of Pharmacy, Health and Nutritional Sciences, University of Calabria, 87036 Rende, Italy; g.bagetta@unical.it; 2 Regional Center for Serious Brain Injuries, S. Anna Institute, 88900 Crotone, Italy; mariannacontrada@gmail.com (M.C.); patonin18@gmail.com (P.T.); 3 Department of Health Sciences, University “Magna Graecia” of Catanzaro, 88100 Catanzaro, Italy; mtcorasa@unicz.it; 4 German Center for Neurodegenerative Diseases (DZNE), 53127 Bonn, Germany; pierluigi.nicotera@dzne.de

**Keywords:** COVID-19, dementia, pain assessment, NPSs, MOBID-2, tele-neurorehabilitation

## Abstract

The coronavirus disease 2019 (COVID-19) pandemic imposes an unprecedented lifestyle, dominated by social isolation. In this frame, the population to pay the highest price is represented by demented patients. This group faces the highest risk of mortality, in case of severe acute respiratory syndrome coronavirus (SARS-CoV-2) infection, and they experience rapid cognitive deterioration, due to lockdown measures that prevent their disease monitoring. This complex landscape mirrors an enhancement of neuropsychiatric symptoms (NPSs), with agitation, delirium and reduced motor performances, particularly in non-communicative patients. Due to the consistent link between agitation and pain in these patients, the use of antipsychotics, increasing the risk of death during COVID-19, can be avoided or reduced through an adequate pain treatment. The most suitable pain assessment scale, also feasible for e-health implementation, is the Mobilization-Observation-Behaviour-Intensity-Dementia (MOBID-2) pain scale, currently under validation in the Italian real-world context. Here, we report the case of an 85-year-old woman suffering from mild cognitive impairment, subjected to off-label treatment with atypical antipsychotics, in the context of undertreated pain, who died during the pandemic from an extensive brain hemorrhage. This underscores the need for appropriate assessment and treatment of pain in demented patients.

## 1. Introduction

The outbreak of coronavirus disease 2019 (COVID-19) imposes novel life rules, dominated by quarantine, social distancing (up to lockdown) and the use of personal protective equipment. The distance and the assistance by mask-wearing, seemingly faceless caregivers, worsen the cognitive and behavioral consciousness and, consequently, affect the most fragile population, i.e., the uncommunicative elderly, suffering from moderate to severe dementia. Currently, some 55 million people suffer from dementia, with 75% demented people (some 41 million) being undiagnosed [1], and this number is estimated to reach 78 million by 2030 and 139 million by 2050. Patients affected by Alzheimer’s disease (AD) have contracted the infection more frequently and present increased risk of death [2,3]. In fact, 80% of COVID-19 deaths are in nursing homes, since living in care facilities increases the risk of mortality [4]; accordingly, more than a third of mortality can be represented by AD patients [5]. The analysis of the primary health records of 13,338 UK individuals shows that a pre-existing diagnosis of AD is associated with the highest risk of COVID-19 and mortality [6]. People suffering from dementia present an increased risk of contracting the infection, due to severe acute respiratory syndrome coronavirus (SARS-CoV-2) and develop more serious sequelae, induced both by severe COVID-19 and by isolation measures [7]. In fact, both conditions can aggravate neuropsychiatric symptoms (NPSs) [8], confusion and agitation [7], because of the interaction with pharmacological treatments [9], and COVID-19 can present as delirium and worsened agitation in these patients [10,11]. However, some disorders, such as Wilson’s disease, were surprisingly not affected by COVID-19 pandemic [12]. Agitation can represent a form of help-seeking in response to unrelieved pain [13,14], one of the triggers commonly responsible for agitation that can be effectively managed through analgesics [15]. In fact, the chronic pain treatment of AD patients is limited in real-world settings and related with increased and harmful use of antipsychotics and antidepressants [16,17,18]. The severity of pain is associated with NPSs and antipsychotics prescriptions [19] and a Delphi consensus proves the priority of analgesia in the management of NPSs [20]. Therefore, an integrated pain management [21], allowing appropriate pain treatment, can reduce the use of antipsychotics for NPSs treatment [22,23], decreasing cardiocerebrovascular events that represent a mortality risk factor for COVID-19 [24]. Almost two years after the onset of this dramatic pandemic, and although lots of documents, produced by several countries, to provide instructions for adequate and supportive care for people with dementia, specific guidelines for pain assessment and management for patients with dementia, also in the circumstance of forced social isolation, are currently unavailable. The evidence gathered prompts the presentation of a case report, pointing at the urgent need for accurate pain assessment and treatment in the fragile population of the elderly, suffering from mild cognitive decline and dementia, with impaired communication capabilities.

### 1.1. Neurobiological Common Ground of COVID-19 and Dementia 

The COVID-19 pandemic raises the most serious consequences in the fragile population of the elderly, finding them often affected by cognitive impairment from dementia, named Alzheimer‘s disease, and related dementias (ADRD) [25]. The latter patients display a higher risk to be infected by SARS-CoV-2 because of the difficulty for them to remember and put into practice the preventative safeguard measures, adopted worldwide, and also due to their dependence on caregivers and assistance in nursing homes, often with sustained virus circulation [25]. Social restriction enhances the already occurring isolation of these patients and necessary effective telehealth programs, to impede the complete loss of motor abilities, are still not adopted in many real-world settings [26]. Incidentally, COVID-19 has been demonstrated to induce neurological symptoms attributable to the central nervous system, e.g., impaired consciousness, but also agitation, confusion and corticospinal tract signs [27]. Cognitive deterioration can be due to the development of acute respiratory distress syndrome (ARDS), which occurs in 14.8% cases [28]. As for AD, anosmia is a frequent symptom and NPSs, delirium and a change in mental health can be the presentation of symptoms of the infection [29]. Furthermore, COVID-19 is suggested to be responsible for long-term neurological *sequelae* and dementia. In fact, SARS-CoV-2 receptor angiotensin-converting enzyme-2 (ACE-2) is expressed by neuronal and glial cells [30]. Using 18F-fluorodeoxyglucose (18F-FDG) positron emission tomography/computerized tomography (PET/CT) brain imaging, in the course of acute encephalopathy, patients displaying cognitive dysfunction and frontal behavioral changes present hypometabolism of the prefrontal cortex, predominant on the right side, bilateral insula, anterior cingulate and caudate nucleus, associated with increased blood (and in some cases cerebrospinal fluid) levels of interleukin-6 (IL-6) [31]. The nucleotide-binding domain and leucine-rich repeat (NLR) pyrin domain-containing 3 (NLRP3) inflammasome, known to be implicated in AD [32,33], is involved in systemic inflammation, induced by SARS-CoV-2 infection [27,34]. This is demonstrated by the evidence of vascular and aberrant immune reaction-related factors [35]. Furthermore, after contracting COVID-19, the risk of being hospitalized is doubled in patients homozygous for APOEε4, a known risk factor for AD [36], that results in a predictor of doubled risk of severe infection and of quadruple risk of mortality [37,38,39]. The so-called, COVID-19-induced “brain fog” can be long-lasting, accelerating cognitive decline in AD patients, and this may be supported by the western blotting evidence of increased levels of ACE-2 in AD patients’ hippocampal tissue [40]. Moreover, brain hypoperfusion can accelerate amyloid-β (Aβ) and tau accumulation, together with the transactivation response DNA-binding protein of 43 kDa (TDP-43) pathology [41].

### 1.2. Observational Pain Assessment

The evidence that patients suffering from dementia develop an aberrant transmission and modulation of pain [42], together with age-related comorbidities associated with pain, accounts for the presence of chronic pain states in 50% demented people [43], reaching 80% in long-term care facilities [44], often under-detected [45,46]. Pain assessment is difficult in patients suffering from severe dementia due to their reduced communication skills. Therefore, behavioral and physiological indicators of pain, in particular pain noises, facial expressions and defensive behaviours, are fundamental to diagnose and unravel non-self-reported pain. Under-treated pain is tightly connected to NPSs, causing agitation, which is managed through neuroleptics, doubling the risk of death for cardiocerebrovascular accidents in the elderly [47], representing a mortality cause during COVID-19 infection; an appropriate diagnosis and therapy for pain is fundamental to reduce the risk of death [24]. A retrospective case-control study provides evidence for increased mortality risk in patients, after initiation of a treatment with an antipsychotic (i.e. haloperidol, olanzapine, quetiapine, and risperidone) [48].

## 2. Case Report

Here, we present the case of an 85-year-old woman, with mild cognitive impairment, who died during the pandemic, due to the lack of accurate pain management in the real-world setting of a Southern Italian health district. The neurologic visit led to the prescription of sertraline and haloperidol, the latter administered during acute attacks. After a fall, causing persistent leg joint pain, the patient was sent for geriatrician evaluation, for worsened cognitive dysfunction and unmanageable agitation. The pain assessment and treatment was delayed because of the health emergency and the agitation was considered so severe as to require institutionalization and off-label treatment with the antipsychotic, quetiapine. Within the first ten days after admittance to the nursing home, the mild cognitive impairment progressed into uncommunicative, rapid cognitive decline, accompanied by intractable agitation and delirium, leading to high dosage benzodiazepines that contributed to exacerbating the confusion. One week later, the patient was not able to recognize her relatives and died from an extensive brain hemorrhage. In fact, she was moved to the emergency room of the referred hospital for suspected stroke, but imaging analysis proved it was a brain hemorrhage that led to her death the following day in the hospital. The patient presented good hematochemistry parameters and she had no other cardiovascular risk factors, apart from treatment with antidepressants, previously. The patient had suffered a previous stroke, earlier than ten years and presented senile atrophy, demonstrated by neuroimaging. Unfortunately, this case confirms that delirium may be associated with an increased risk of hospitalization and mortality [49]. Particularly remarkable, is that the cognitive and neuropsychiatric status precipitated due to the lack of adequate pain assessment and treatment.

## 3. Discussion

### 3.1. Pain Scales and E-health 

Pain assessment is fundamental to improve the quality of life of patients suffering from dementia and to decrease their mortality risk during the pandemic. One of the barriers most difficult to overcome in dementia is represented by the loss of communication skills that these patients experience, preventing them from pain self-report. Furthermore, the social isolation contributes to the worsening of cognitive impairment, and the delaying of non-urgent medical examinations causes the neglection of the assessment, monitoring and treatment of dementia [50] and consequently pain and NPSs. Furthermore, the lack of care exacerbates the reduction in motor function of cognitively impaired patients, requiring rehabilitation. Therefore, observational pain scales, feasible for telehealth administration, are needed during the pandemic. In fact, tele-neurorehabilitation has proven not inferior to standard treatment [51]. Among the existing internationally validated observational pain scales for uncommunicative patients affected by severe dementia, the Mobilization-Observation-Behaviour-Intensity-Dementia (MOBID-2) pain scale is unique of its kind, being developed to take into account the common overlapping of musculoskeletal and visceral pain [52], through behavioral indicators of pain, i.e., pain noises, facial expressions and defensive behaviours. The first part of MOBID-2 was developed for the assessment of musculoskeletal pain, also unraveling hidden pain conditions, through five guided movements of different body parts [53]. The second part is developed for the assessment of pain from internal organs, head and skin [53]. The international validation of this tool in severe dementia highlights its remarkable psychometric properties, as good face and construct (Part1 rho = 0.82; Part2 rho = 0.61) validity and very good inter-rater (ICC (1, 1) 0.80–0.94) and test–retest (ICC (1, 1) 0.60–0.94) reliability (Table 1) for pain intensity [53]. This scale is suitable for virtual assessment of pain and its Italian version is currently under validation in the Italian context. 

### 3.2. The Possible Role for E-health

In the initial phases, Italy was overwhelmed by the pandemic, with 11.9% of deceased COVID-19 positive patients affected by dementia [54], afflicting 900–1000 per 100,000 inhabitants [55]. Particularly worthy of note is that demented patients are more at risk to receive inadequate treatments during the health emergency [54]. Unfortunately, the latter observation finds confirmation in pain management during the pandemic. The overlapping of several pathogenetic pathways, between AD and COVID-19, accounts for their synergistic action in cognitive deterioration, enhancing the frailty of patients suffering from dementia in this scenario. Lockdowns and social distancing worsen the cognitive and motor performances of these patients and delay their monitoring, making it necessary to provide severely demented patients with pain assessment and neurorehabilitation by means of telemedicine. An excellent example to follow is represented by the development of intelligent assistive systems, to obtain high-quality behavior data from real-world environments [56]. Here, we report the case of an 85-year-old woman, suffering from mild cognitive impairment, who died from an extensive brain hemorrhage during the last period of the health emergency, because of the lack of accurate pain management, leading to institutionalization and off-label treatment with atypical antipsychotics, known to double the risk of death of these fragile patients. Although the study limitation that an actual causal relationship cannot be established, this case report supports the need for novel efficient strategies, including telemedicine, to afford appropriate assessment and treatment of pain in demented patients during the pandemic.

## 4. Conclusions: Novel Pharmacological Perspectives

The tight link between COVID-19 and dementia highlights the need for greater attention to pain assessment and treatment during the pandemic. Dementia can be exacerbated by acute respiratory distress syndrome, the neurological sequelae of the infection and, also, the social isolation measures to prevent contagion. In particular, the highest mortality for COVID-19 is registered among patients suffering from dementia that are often diagnosed with the infection because of a worsening of agitation, one of the most challenging behavioural and psychological symptoms of dementia (BPSD), and the occurrence of delirium. Agitation is often a form of help-seeking for underdiagnosed and, consequently, unrelieved pain, in non-communicative demented patients. The assessment and management of chronic pain, often experienced by patients affected by dementia due to age-related comorbidities, is made even more difficult during the pandemic because of lockdowns and delayed specialistic examinations. This complex scenario leads to an increased rate of inappropriate prescriptions of antipsychotics to manage agitation due to undertreated pain, doubling the risk of death from stroke, which can be a complication of COVID-19, in the fragile population of demented patients. Therefore, it is fundamental to avoid the unnecessary and harmful use of antipsychotics and antidepressants; on the other hand, the novel rapid-acting antidepressants are worthy of investigation [57]. This paves scenario the way for the use of essential oils, also feasible in telemedicine healthcare; among these, the essential oil of bergamot is the most suitable for its proven analgesic [58] and anxiolytic-like properties [59]. Moreover, it has been developed in the form of a cream, carrying a nanotechnological delivery device to preserve the oil and allow double-masking [60] of the ongoing clinical trial, for proof of concept in severe dementia (NCT04321889) [61]. This can allow adequate pain management, avoiding or reducing, also, the use of potent analgesics, such as opioids, endowed with serious side effects and not properly tested for efficacy and safety in fragile populations, including dementia-affected and post-stroke patients [62].

## Figures and Tables

**Table 1:** Clinimetric properties of the Mobilization-Observation-Behaviour-Intensity-Dementia (MOBID-2) pain scale.

Description of the Pain Assessment Tool	Structure of the Scale	Time Efficiency	Healthcare Operator in Charge	Psychometric Properties: Validity and Reliability
Observational scale for non communicative patients affected by severe dementia	It is composed by 10 items, 5 per each part. The part 1 is conceived for the assessment of musculoskeletal pain through the guided execution of 5 active movements to unravel also hidden pain conditions. In the part 2 pain from head, skin and internal organs is assessed.	The mean time needed for execution is 4.37 min.	Nurses receiving a 2 h educational session.	Inter-rater and test–retest reliability for pain intensity: ICC 0.80–0.94 and 0.60–0.94. Correlation with physicians’ numerical rating scale (NRS) scoring and defined pain variables (rho = 0.41–0.64) [53].

## Data Availability

Data is contained within the article.
